# Light controlled assembly of silver nanoparticles

**DOI:** 10.1038/srep45144

**Published:** 2017-03-23

**Authors:** Andreas Polywka, Christian Tückmantel, Patrick Görrn

**Affiliations:** 1 Chair of Large Area Optoelectronics, University of Wuppertal, 42119 Wuppertal, Germany

## Abstract

Metal nanoparticles show a particularly strong interaction with light, which is the basis for nanoparticle plasmonics. One of the main goals of this emerging research field is the alignment of nanoparticles and their integration into sophisticated nanostructures providing a tailored interaction with light. This assembly of nanoparticles at well-controlled substrate sites often involves expensive technological approaches, such as electron beam lithography in order to fabricate the nanoparticle structures. Furthermore difficult numerical simulations are needed to predict their optical properties. Both requirements, fabrication and prediction, complicate a cost-efficient exploitation of nanoparticle plasmonics in optoelectronic devices. Here we show that silver nanoparticles deposited under exposure to visible light arrange in a way that the resulting structure shows an optimized interaction with that light. This way, the light not only controls the nanoparticle alignment with an estimated accuracy of well below 20 nm during deposition from the liquid phase, but also defines the optical properties of the growing structure, and therefore complicated prediction is not needed.

Nanoparticle plasmonics enables a plethora of applications, such as surface enhanced Raman spectroscopy^1^, biological detection^2^,^3^ or light trapping in solar cells^4^,^5^. However, such applications require accurate positioning of high quality nanoparticles.

On the one hand, top-down lithographic approaches like electron beam lithography or focused ion beams enable accurate control over the positions of nanoparticles on a solid substrate^6^. On the other hand, bottom-up synthesis of nanoparticles from solution enables much better control of the nanoparticles on the atomic scale, including crystallinity, size, roughness and shape^7^,^8^. The challenge here is to assemble the nanoparticles, which are randomly distributed in solution, into well-defined nanostructures. While many approaches have been developed that enable self-assembly into certain geometries^9^,^10^,^11^, well-controlled assembly at arbitrary substrate positions is still not possible.

The technique presented here is based on silver nanoparticles (AgNPs) prepared from solution that grow at the interface between the solution and the substrate at a well-controlled position. Recent work has demonstrated how to reach optimized nanoscale morphology of single AgNPs from solution^12^,^13^. In contrast, this paper focuses on the question of how to control the positions where such AgNPs grow on a solid substrate in order to reach an ensemble of optimized optical functionality. The approach therefore combines the accurate positioning of top-down approaches with the benefits of bottom-up fabrication, with an emphasis on facile processing. Instead of expensive equipment, only a beaker for the chemicals, the chemicals themselves and a light source are needed. Without clean room conditions NP positions can be controlled in a scalable approach.

AgNPs are grown by electro-less deposition (ELD)^14^,^15^. The method is slightly modified in order to enhance its light sensitivity and used under light exposure (see Methods and Supplementary Information). As light sources lasers of different wavelength *λ*_0_, incident angle *θ*_*i*_ and polarization (p or s) are used so AgNPs are predominantly deposited at the illuminated substrate sites. Importantly, the nanoscale alignment at these sites strongly depends on the mentioned laser parameters.

## Results

### Incident electrical field dominated by the normal component *E*_*z*_

**Figure 1:**
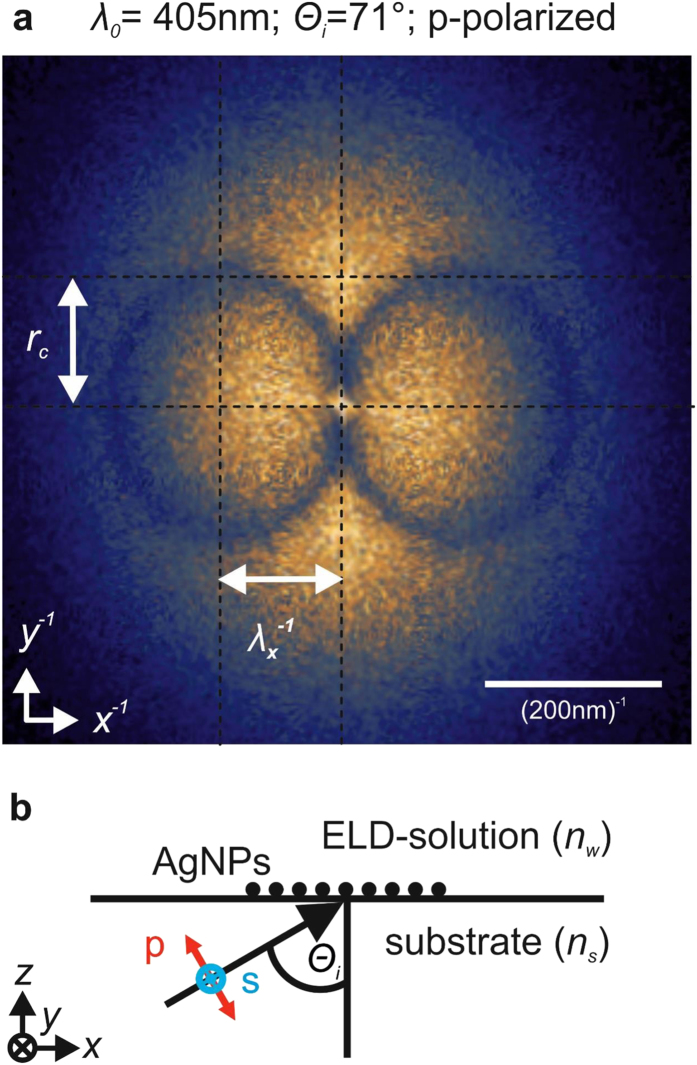
Fourier transformed electron micrograph (FTEM). (**a)** FTEM of a AgNP structure produced under exposure to a p-polarized violet laser beam (*λ*_0_ = 405 nm) incident under *θ*_*i*_ = 71°. (**b)** geometry of the setup: substrate plane: *xy*, plane of incidence: *xz*, polarization p (parallel) and s (perpendicular) to plane of incidence, definition of angle of incidence *θ*_*i*_.

Figure 1a shows the Fourier transformed electron micrograph (FTEM) of a AgNP structure produced with that method. While the electron micrograph itself does already contain the information about the positions of thousands of AgNPs, the Fourier transformation enables systematic analysis of these positions [see also Supplementary Information]. Fourier transformation is a well-established method to analyse morphology^16^,^17^

Particularly noticeable in the FTEM of Fig. 1a are the two (black) circles symmetrical to the vertical centre line (*x*^−1^ = 0). To prepare this structure a p-polarized laser beam of *λ*_0_ = 405 nm and a large *θ*_*i*_ = 71° is chosen in order to ensure that the electric field inside a AgNP is dominated by its *z*-component (see Fig. 1b). Without lateral field components, it is assumed that the excitation of delocalized surface plasmons (SPs) can be neglected because it requires lateral fields^18^. In that case each AgNP can be considered as an independent localized source of light scattering.

In fact the circles found in the FTEM can be directly attributed to the electromagnetic wave scattered by the AgNPs. Propagating in the water-based ELD-solution (refractive index *n*_*w*_) within the substrate plane *xy*, it can be described as a spherical wave. The Fourier transformation (FT) of that wave is a circle with the radius


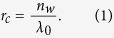


For perpendicular incidence (*θ*_*i*_ = 0) a superposition of many such waves scattered at different substrate position still results in the same FT. On the other hand, the phase of a laser beam exciting the substrate plane under the angle *θ*_*i*_ > 0 is a sinusoidal function of *x* with the period *λ*_*x*_. If many spherical waves are excited by such a coherent beam the resulting FT contains the convolution of the described FT of a spherical wave and the FT of the sinusoidal function (for more details see Supplementary information). As a result the circle representing the scattered waves is shifted by 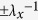 with


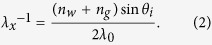


The equations (1) and (2) describing scattering of light also accurately describe the size and position of the circles found in the FTEMs at any *λ*_0_ and *θ*_*i*_. This is remarkable because there is no light scattering present during electron microscopy, nor can electron microscopy detect light. Therefore, we conclude that the electromagnetic waves that were present during fabrication of the structure are stored in the form of the AgNPs positions.

To verify this idea we have developed an algorithm that simulates the particle alignment process. First the average brightness distribution of the AgNPs is determined from the real electron micrograph representing ideal AgNPs. Those ideal particles are placed on a map creating an artificial micrograph. After placing a first particle, its random position is considered the origin of a scattered wave excited by a coherent wave. The local intensity is then calculated from the superposition of all present electromagnetic waves. Afterwards the next particle is placed at the position of maximum intensity under consideration of a standard deviation in the planar directions (Δ*x*, Δ*y*). The algorithm continues until up to 6000 particles, each one being the origin of a scattered wave, are placed in an area of 12 × 12 μm^2^. One has to keep in mind that this large number of particles can be handled only because delocalized SPs have been neglected. The algorithm does assume perfect 2D growth of AgNPs on the substrate surface. This appears realistic as samples with clear FTEM features show only slight 3D packing of AgNPs. The Python code and a video showing the simulated alignment of the particles are included in the Supplementary Information.

**Figure 2:**
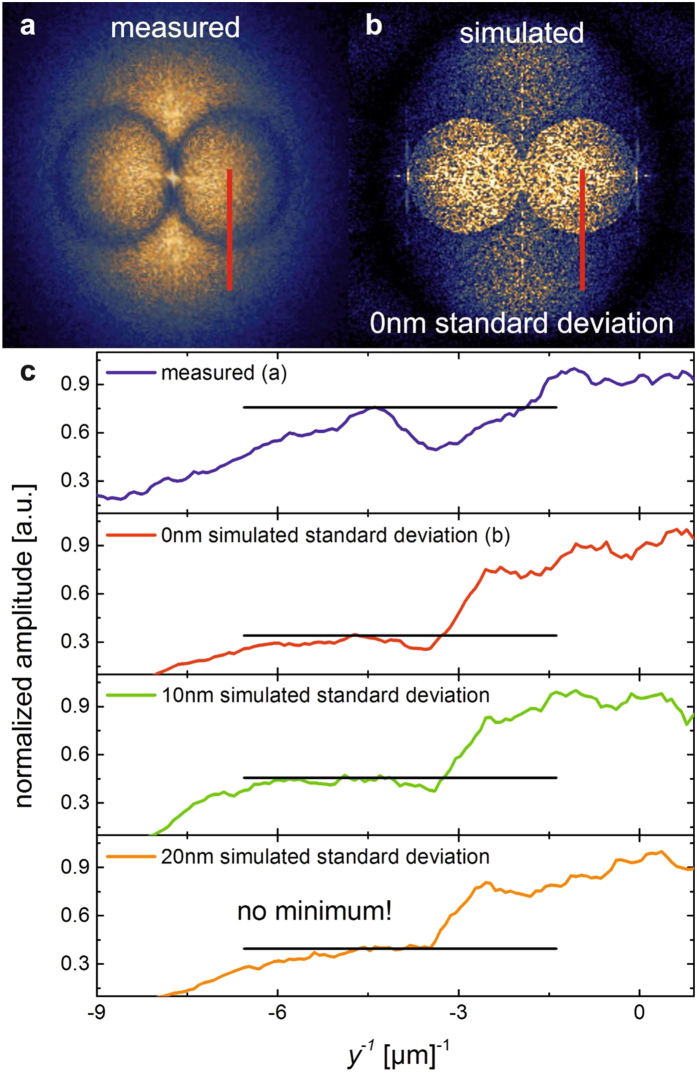
Simulation of AgNP alignment. (**a)** measured FTEM (see Fig. 1a). (**b)** simulated FTEM with identical laser parameters and a standard deviation of zero (accurate placing of the AgNPs). (**c**) FTEM amplitudes at the identical trace (red line in **a**,**b**) highlighting the shape of the circle minimum: with increasing standard deviation the simulated circle minimum disappears.

Figure 2 shows the resulting simulated FTEM in comparison with the measured FTEM. As the incident wave and the scattered ones are in counter-phase, the circles described by equations (1), (2) form a minimum which strongly depends on the standard deviation. Figure 2c shows the shape of the circle minimum for Δ*x* = Δ*y* of zero (red curve and Fig. 2b), 10 nm (green curve) and 20 nm (orange curve). While perfect alignment creates a minimum similar to the experimentally observed one, at Δ*x* = Δ*y* = 20 nm, no minimum is found. This result hints to a positioning accuracy of well below 20 nm.

The simulation also explains why the electromagnetic waves responsible for the alignment are visualized by the FTEMs. AgNPs are always placed at the position of maximum field intensity – in this way storing that information and increasing light scattering of the incident light. This phenomenon can even be observed with the naked eye (see Supplementary Information).

The simulation is able to precisely describe the AgNP alignment considering only localized SPs. We interpret this fact as a confirmation for the assumption that the alignment caused by an electrical field oriented normal to the sample plane is dominated by localized SPs.

### Incident electrical field with lateral (*E*_*x*_, *E*_*y*_) and normal (*E*_*z*_) components

**Figure 3:**
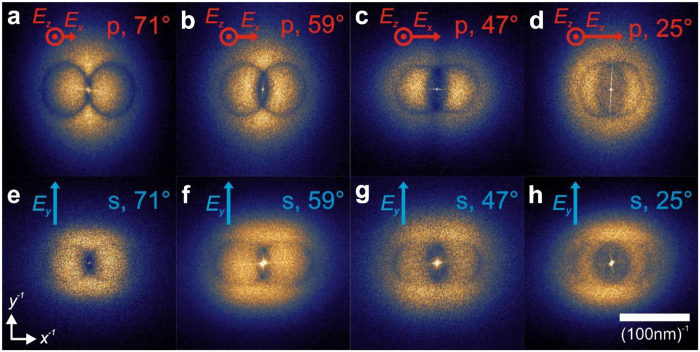
The influence of polarization and angle of incidence. FTEMs of structures prepared with a violet laser beam (*λ*_0_ = 405 nm) incident under different polarization and angle *θ*_*i*_. p-polarization and 71° (**a**), 59° (**b**), 47° (**c**) and 25° (**d**); s-polarization and 71° (**e**), 59° (**f**), 47° (**g**) and 25° (**h**); (FTEMs of structures prepared with different laser wavelength are shown in the Supplementary Information).

Figure 3 shows experimentally obtained FTEMs covering a large range of *θ*_*i*_ below and above the critical angle for total reflection (*θ*_*c*_ = 65°). For p-polarization (Fig. 3a...d) with decreasing angle *θ*_*i*_ the circles become more and more fuzzy. That indicates that localized SPs still contribute to the alignment, but their impact decreases with increasing lateral field *E*_*x*_. In the case of s-polarization (Fig. 3e...h), the lateral electrical field reduces the influence of localized SPs at any *θ*_*i*_. Furthermore with decreasing angle two maxima occur that are separated in the reciprocal direction of the lateral electrical field. These maxima cannot be explained with localized SPs and cannot be simulated with the approach described above. The influence of circles and maxima on the FTEMs increases with the normal or lateral field component, respectively. This finding hints at a coexistence of localized and delocalized SPs^19^. While the circles represent localized SPs, we conclude that the maxima may represent delocalized SPs. To investigate that phenomenon perpendicular incidence is used.

### Perpendicular incidence with lateral (*E*_*x*_) components, only

**Figure 4:**
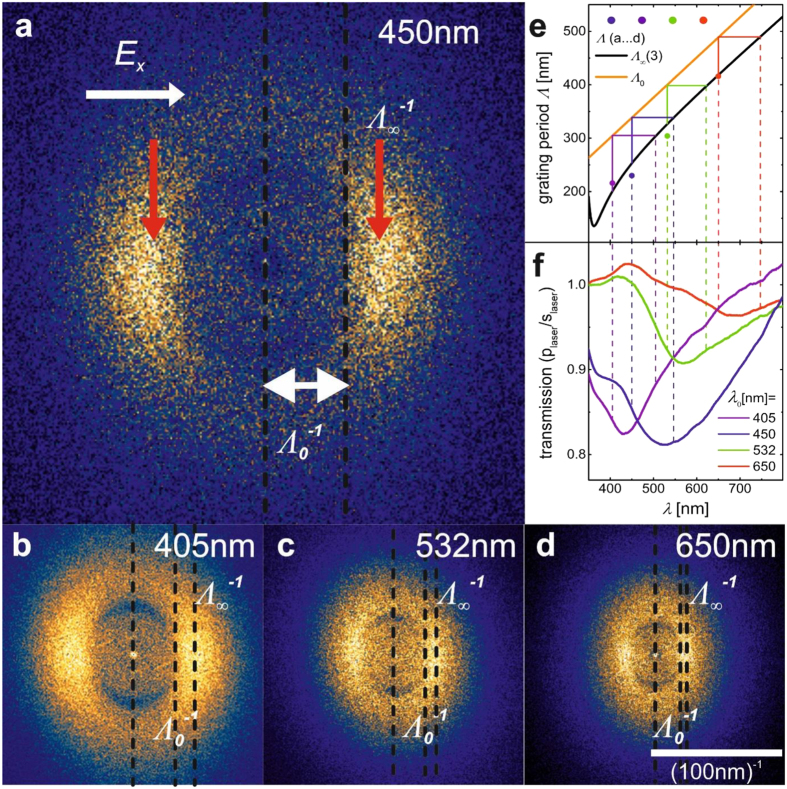
Perpendicular incidence – the preparation of AgNP gratings exciting delocalized SPs. FTEMs of structures prepared at *λ*_0_ = 450 nm (**a**), 405 nm (**b**), 532 nm (**c**), 650 nm (**d**). (**e**) measured grating periods Λ (maximum intensity in FTEMs (**a**…**d**) shown as coloured points in comparison to the boundaries Λ_0_ and 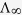 (also shown in (**a**…**d**); the variation of Λ (vertical line) is translated to the wavelength scale for comparison with **(f**); (**f**), normalized transmission spectra (*p*_*laser*_/*s*_*laser*_; *p*_*laser*_ = polarization parallel to laser, *s*_*laser*_ = polarization perpendicular to laser) of the structures (**a…d)**.

For perpendicular incidence, a distinction between s- and p-polarization is not needed anymore. Figure 4 shows the corresponding FTEMs for different laser colours *λ*_0_. With 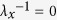 (2) only one centred circle remains that is caused by scattering at localized SPs. However, as discussed above the alignment process is now assumed to be dominated by coupling of localized SPs to delocalised SPs. A theoretical description of this coupling phenomenon within a structure consisting of thousands of AgNPs is quite unthinkable.

On the other hand, the FTEMs again enable visualization of the electromagnetic phenomena responsible for the alignment. As indicated by the vertical maxima (red arrows in Fig. 4a) the AgNPs now align into a grating structure of period Λ, at the same time visualizing a delocalized SP wave of the wavelength *λ*_*sp*_ = Λ propagating in the direction of the electrical field that is excited by the grating^20^:


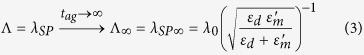


For thick silver films (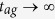) a SP wavelength 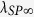 is presented in the literature (right equation (3)). The maximum intensity in the FTEMs fits quite well with this dispersion relation of SPs at the glass/silver interface (see Fig. 4e). This confirms that the maxima in the FTEMs visualize not only the gratings able to excite delocalized SPs, but at the same time visualize the delocalized SPs themselves. Being longitudinal waves they are excited only when the field of the incident wave is oriented in the direction of the gratings’ wave vector^18^,^21^. This is also the direction of the SP propagation. It confirms the assumption that lateral fields support AgNP alignment mediated by delocalized SPs.

However, one has to keep in mind that the propagation of delocalized SPs requires a significant mass thickness of the nanostructure. At the beginning of the AgNP deposition lateral waves will be excited by scattering of localized SPs, only. These waves will predominantly propagate in water. Hence, the grating period defined during deposition ranges from 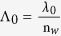 (the wavelength of the laser light in water) to 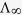. This variation of grating periods can easily be observed in the FTEMs in Fig. 4a...d. It is highlighted in the vertical solid lines in Fig. 4e and translates into a variation of the wavelength of optimized interaction (horizontal lines in Fig. 4e). This wavelength range can be found in relative transmission spectra of the structures with the FTEM shown in 4a…4d. The extinction is increased for light that shows the polarization and colour of the laser beam used to align the structure.

## Discussion

It is therefore a general observation that the reported AgNP alignment leads to a maximized interaction with the wave controlling that alignment. Without lateral field components (Fig. 3a), the scattering at localized SPs is maximized. If lateral field components exist, a grating for optimized excitation of delocalized SPs is created. In the general case, the FTEMs reveal a combined excitation of localized and delocalized SPs.

Beyond this self-optimization of optical properties, the presented technique may find applications in nanotechnology. While in the presented approach only one light source and a plane substrate without any patterning were used, clear boundary conditions for the involved electromagnetic waves may enable a more direct control of the patterns. Such boundary conditions may include metal frames, plasmonic or dielectric waveguides, resonators, patterned gain media and others on the substrate. Instead of one laser, different light sources interfering with each other may be applied. With the capability of plasmonics to guide and focus electromagnetic waves to the nanoscale accurate assembly of nanoparticles may result.

In summary, we have shown that AgNPs can be aligned by light. During that alignment process, every nanoparticle grows at the position of maximum local intensity. This position of course changes with every new nanoparticle. In this way, a feedback between light influencing the nanostructure and the nanostructure influencing the light is created. As a consequence, the incident light beam leaves a unique fingerprint on the structure. This not only causes an optimized interaction of the AgNP structure with that light, but also enables a way to visualize the local electromagnetic phenomenon caused by the light inside the nanostructure (e.g. scattering at localized SPs or excitation of delocalized SPs). Localized SPs are represented by circles in the FTEMs. Their impact on the alignment increases with the normal field component of the incident wave. By contrast, delocalized SPs represented by two maxima in the FTEM dominate under lateral field incidence. We anticipate that the presented feedback for tailoring plasmonic nanostructures will open up new paths in both fields: optics and nanotechnology.

## Methods

### Sample preparation

A glass substrate (size 17 × 17 mm^2^) is placed on a half-circular prism with index matching liquid in between. A laser is aligned so its beam hits the substrate surface under a certain angle and polarization. The power density was always adjusted to about 140 mWcm^−2^. An additional wire grid polarizer was placed in the laser beam. After the laser is switched on, about 0.5 cm^3^ of the solution 20 A (see Supplementary Information) is cast on the substrate. After 10 minutes the deposition of AgNPs is stopped by placing the substrate in DI water. After a longer deposition time, the film thickness becomes too high and intense 3D packing of nanoparticles is observed. In that case, the features in the FTEM become fuzzy and even disappear at very large thicknesses.

### Electron microscopy

The AgNP nanostructure was investigated using a Philips XL30S FEG microscope with a field emission cathode.

### Optical Spectroscopy

Transmission spectra were determined using a white deuterium halogen lamp (DH-2000-BAL, 800 μWcm^−2^, OceanOptics) and a spectrometer with a range from 186 to 1041 nm (USB 2000 + XR1-ES), respectively.

## Additional Information

**How to cite this article:** Polywka, A. *et al*. Light controlled assembly of silver nanoparticles. *Sci. Rep.* **7**, 45144; doi: 10.1038/srep45144 (2017).

**Publisher's note:** Springer Nature remains neutral with regard to jurisdictional claims in published maps and institutional affiliations.

## Supplementary Material

Supplementary Information

Supplementary Video 1

Supplementary Video 2

Supplementary Video 3
