# Supplementary material for: Light controlled assembly of silver nanoparticles
Source: Sci Rep. 2017 Mar 23;7:45144. doi: 10.1038/srep45144 (PMC5362827; doi:10.1038/srep45144)
Supplement: Supplementary Information [file srep45144-s1.pdf]

Supplementary Information

Light controlled assembly of silver nanoparticles.

*Andreas Polywka, Christian Tückmantel, and Patrick Görrn**

1. Deposition method and Setup

The mixture of chemicals used for electroless deposition of AgNPs is termed ELD solution. It is based on the solution described by Saito et al., but modified similarly to the description in Polywka et al. (step 4)[1,2]. The more ammonia solution is added in step 4, the more the parasitic deposition at dark substrate sites is suppressed. However, the reaction speed at the illuminated sites is also decreased. As a trade-off, the following recipe was used allowing a reaction time of 10 minutes.

1. A 30 wt % ammonia solution is added to 3mL of 2 wt% silver nitrate aqueous solution until the mixture begins to turn brown due to the formation of a precipitate of Ag_2O and then turns clear again as the precipitate completely dissolved to form $[Ag(NH_3)_2]^+$.
2. Then 6 wt % silver nitrate solution is added until the solution becomes pale brown/yellow.
3. 6 wt % ammonia solution is added until the solution becomes transparent again (typically 2 to 3 drops).
4. <u>Modification for increase of the light sensitivity and decrease of the reaction speed:</u> An additional 20 drops of 6 wt % ammonia solution are added in order to increase the light sensitivity.
5. This [Ag(NH3)2]+ solution is mixed with 1 mL of 35 wt % glucose solution.
6. This ELD solution is cast on top of the substrate.

The deposition setup is shown in Figure S1. The setup ensures that the external angle is identical to the internal angle θ_i under which the laser beam hits the substrate surface (see Fig. 1b). At this

surface the ELD solution is cast after the laser has been switched on. After 10 minutes the substrate is removed from the Index matching liquid (IML) and put in a H_2O stop bath in order to halt deposition.

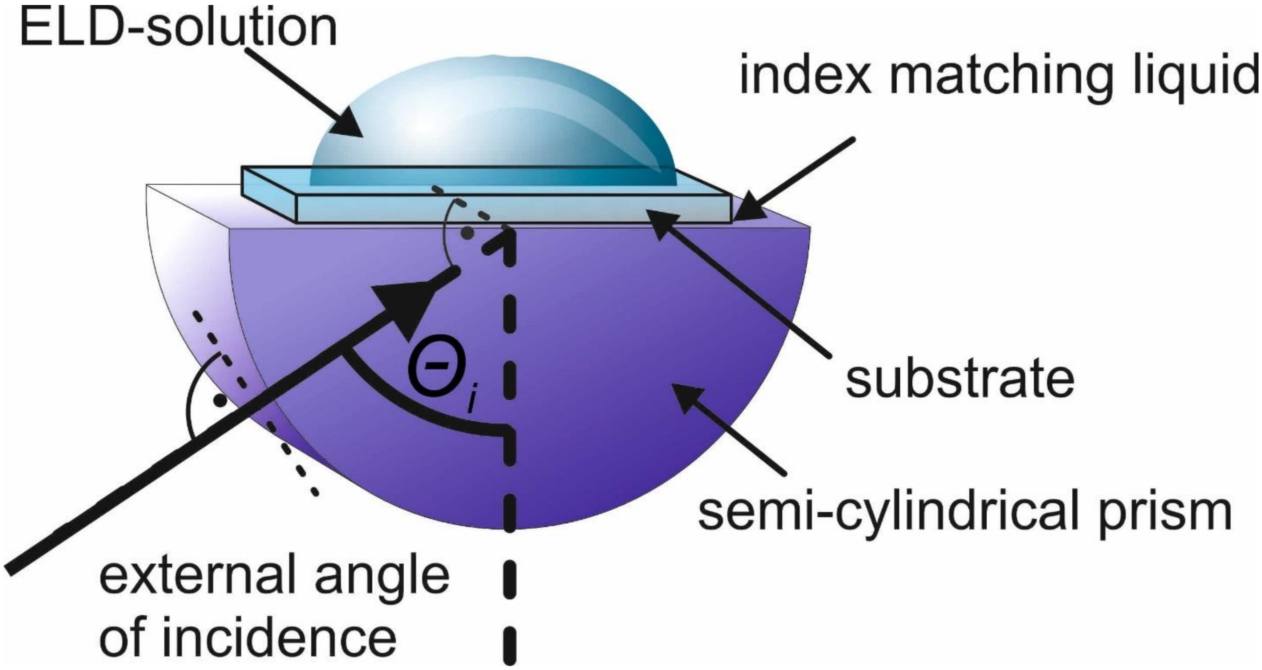

Figure S1 Experimental Setup. Index matching liquid (IML) is placed on a semi-cylindrical glass prism and the glass substrate is placed on the IML. Substrate, IML and prism have the identical refractive index. Air bubbles in the IML are avoided. The laser beam is focused to the centreline of the prism at perpendicular incidence to that centreline. The ELD solution is cast on top of the substrate.

The light sensitivity of the presented approach enables macroscopic patterning as shown in Figure S2. The focus of our paper, however, is on nanoscale assembly of nanoparticles.

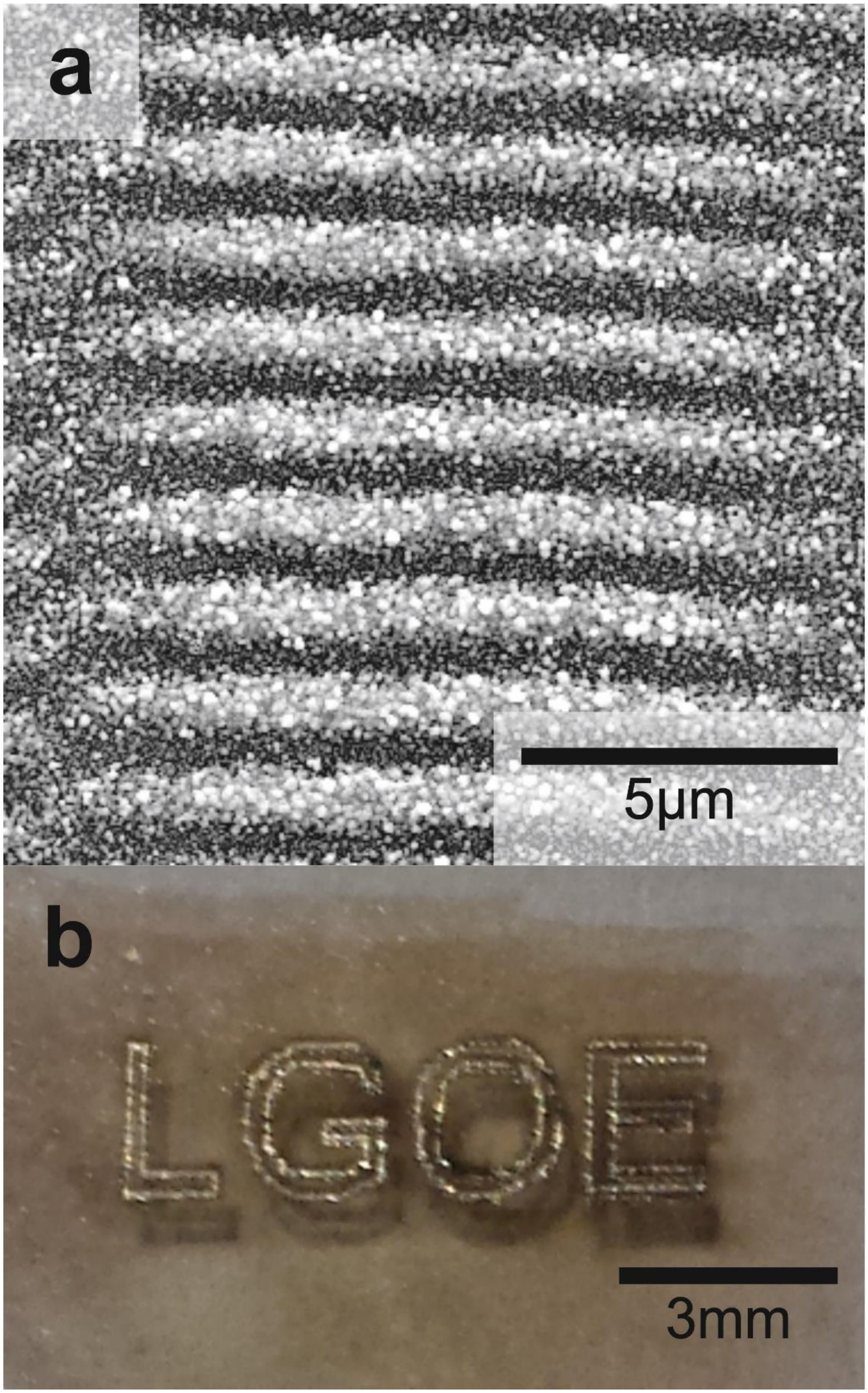

Figure S2 Light sensitivity enables macroscopic patterning. a, modification of the local thickness caused by interference of the incident laser. **b,** macroscopic patterning of the AgNP film using laser gravure system.

2. Selected electron micrographs

Figure S3 presents electron micrographs and Fourier transformed electron micrographs not included in the manuscript. S3a shows a AgNP grating formed during perpendicular incidence (compare Figure 4). In the vertical direction, an increased coalescence of particles is found while the periodic separation in the horizontal direction enable the excitation of delocalized SPs. On the contrary, p-polarised lasers under large incident angle lead to maximized scattering of localized SPs (Figure S3b, compare Figure 1). Consequently, the grown particles are clearly separated in all directions and show crystalline shape.

The figures S3c and S3d visualize the impact of the wavelength under such parameters (p-polarization, large Θ_i). With increasing wavelength, the particles become bigger. This is in agreement with reports about the resonant wavelength of nanoparticles increasing with the particle size[3]. This results in a smaller FTEM. At the same time the circle radii become smaller as predicted by equation 1.

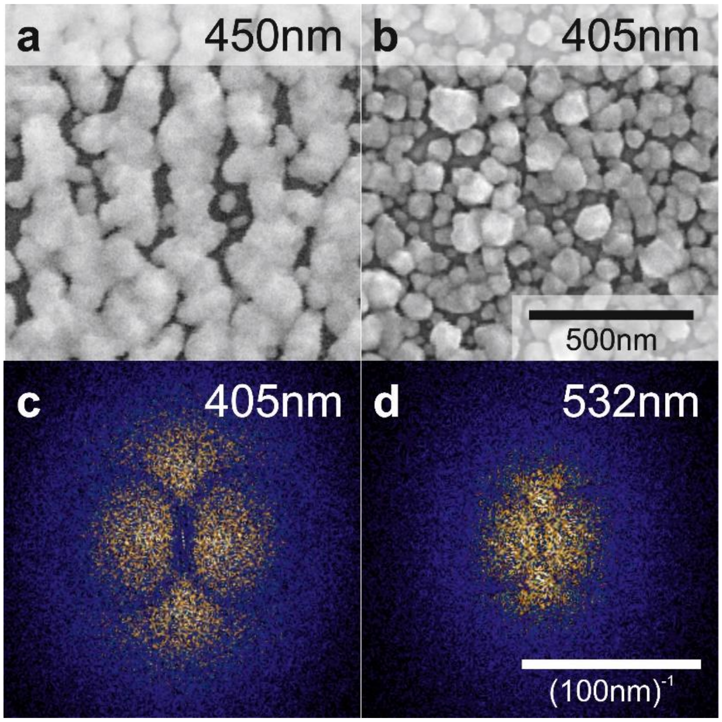

Figure S3 Electron micrographs and Fourier transformed electron micrographs. a, typical electron micrograph of a AgNP (grating) structure prepared at λ_0=450nm and perpendicular incidence. **b,** electron micrograph of a structure prepared with λ_0=405nm, Θ_i=71°, emphasizing the crystalline nature of the AgNPs. **c,d** showing the impact of λ_0 on the FTEM (here Θ_i=71°): with increasing λ_0 FTEM size decreases (particle size increases) and the circle diameter also decreases (equation 1).

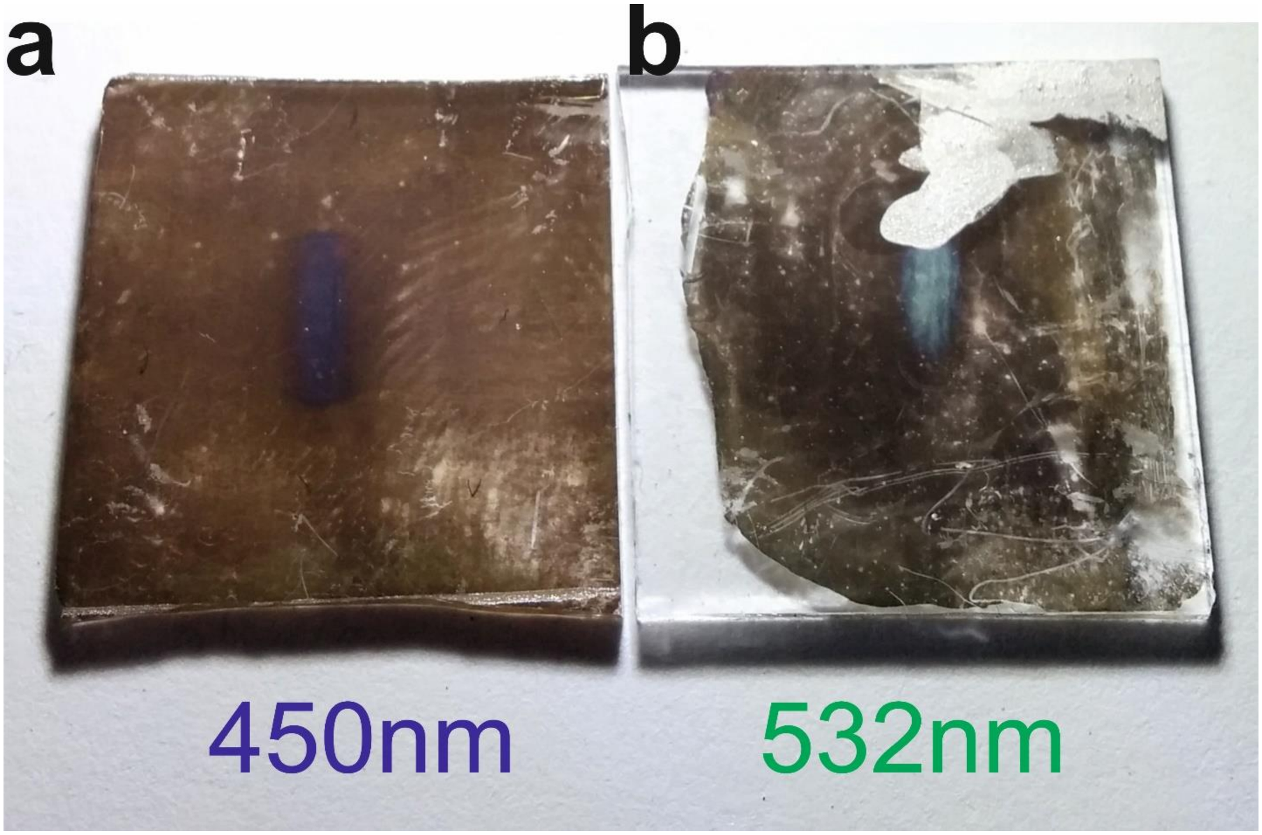

Figure S4 Photographs showing optical scattering of the samples. a, λ_0=450nm **b,** λ_0=532nm: The scattered light shows the colour of the laser beam incident during AgNP deposition. Outside the exposed area a thin parasitic layer can be observed.

Figure S4 shows the scattered light of AgNP nanostructures grown with a blue and green laser, respectively. Under white illumination it can easily be observed with the naked eye that the colour of the laser defines the colour that is scattered most efficiently.

3. The simulation of AgNP alignment

One particle at x_i, y_i is described by a brightness distribution function $\mathrm{b}_i(x - x_i, y - y_i)$ that represents the real electron micrograph of a single AgNP in the substrate plane x, y with

$r(x, y) = \sqrt{x^2 + y^2}$.

The maximum brightness (1) according to the maximum local silver thickness is expected in the centre of the particle ($x - x_i = 0$ and $y - y_i = 0$). Outside the particle ($r(x - x_i, y - y_i) > r_{particle}$) no material is detected and hence the value 0 is expected.

$$\mathrm{b}_i(x - x_i, y - y_i) = \begin{cases} \left(1 - \sin\left(\frac{\pi}{2r_{particle}} r(x - x_i, y - y_i)\right)\right) \mathrm{e}^{wr(x-x_i,y-y_i)} & 0 \le r(x - x_i, y - y_i) \le r_{particle} \\ 0 & r_{particle} < r(x - x_i, y - y_i) \end{cases}$$

Here $r_{particle}$ is the radius of the particles. The exponential function $\mathrm{e}^{wr(x-x_i,y-y_i)}$ allows one to tune the edge of the particle. Both parameters $r_{particle}$ and w are chosen to best represent real measurements.

The particles are excited by a plane wave $\boldsymbol{E_{pw}}$. The z-component of that wave ($E_{pw,x} = E_{pw,y} = 0$) is:

$$E_{pw,z}(x) = \mathrm{Re}(A_{pw} e^{-j(k_x x)}).$$

The scattered wave (spherical wave) within the substrate plane x, y is defined as follows:

$$E_{sw,z,i}(x - x_i, y - y_i) = \begin{cases} 0 & 0 \le r(x - x_i, y - y_i) \le r_{particle} \\ \mathrm{Re}\left(A_{sw} \underbrace{e^{jk_w r(x-x_i,y-y_i)}}_{\text{spherical wave}} e^{-j(k_x x_i)} \frac{r_{particle}}{r(x - x_i, y - y_i)}\right) & r_{particle} < r(x - x_i, y - y_i) \end{cases}$$

with the phase of the exciting plane wave $-k_x x_i$ and the phase of the excited spherical wave $k_w r(x - x_i, y - y_i)$.

The resulting electrical field caused by the scattering of the plane wave can be calculated according to Kelly et al. and Amendola et al. [4,5]:

$$E_{res,z} = E_{pw,z}(x) - E_{total,z}(x, y)$$

$$E_{total,z}(x,y) = \sum_{i=1}^{N} E_{sw,z,i}(x - x_i, y - y_i)$$

With N as the number of particles that are placed. Note that in the algorithm $E_{total,z}(x,y)$ is normalized in order to ensure that the scattered intensity is below the incident intensity.

From that electrical field the intensity can be calculated as follows:

$I(x,y) \propto \left|E_{pw,z}(x) - E_{total,z}(x,y)\right|^2$.

This intensity function is used in order to define the positions x_i, y_i, where the particles are placed.

The simulation starts by placing a particle at a random position $x_1 = x_{ran}, y_1 = y_{ran}$. The initial simulated brightness distribution $\mathrm{b}_{total}(x,y)$ is defined:

$$\mathrm{b}_{\mathrm{total}}(x,y) = \mathrm{b}_1(\mathrm{x} - \mathrm{x}_1, \mathrm{y} - \mathrm{y}_1)$$

$I(x,y)$ is then calculated with this one scattering particle only.

The maximum intensity $I_{max,1}$ outside the particle (later outside all existing particles) is determined. The position (x_2, y_2), where $I_{max,1}$ is found, is stored. A new particle is placed with its centre at this position by adding $b_2(x - x_2, y - y_2)$ to $b_{total}(x,y)$. Outside these two particles a new (typically smaller) maximum intensity $I_{max,2}$ is found. If $I_{max,2} > i_{min} \cdot I_{max,1}$ a new particle is placed at x_3, y_3. This procedure ends when $I_{max,n} < i_{min} \cdot I_{max,1}$.

Then the new $I(x,y)$ is calculated considering all scattering particles. The procedure is concluded when the maximum number of particles N is reached. The simulated electron micrograph is then defined by:

$$\mathrm{b}_{\mathrm{total}}(x,y) = \sum_{\mathrm{i}=1}^{\mathrm{N}} \mathrm{b}_{\mathrm{i}}(\mathrm{x} - \mathrm{x}_{\mathrm{i}}, \mathrm{y} - \mathrm{y}_{\mathrm{i}})$$

List of parameters:

variable	explanation	value*
a_{area}	the length of the quadratic area that is simulated	12µm
dim	number of pixels in the per a_{real}	2400
$r_{particle}$	particle radius	110nm
w	defining the edge of the particles	$0.016nm^{-1}$
err	Standard deviation of the particle position	0nm
i_{min}	Defining how many particles are placed before the next determination of the local intensity (typically 80%...100%)	80%
A_{pw}	normalized amplitude of the plane wave	1
A_{sw}	normalized amplitude of the scattered field	1
λ_0	vacuum wavelength	405nm
Θ_i	angle of incidence	70°
n_{eff}	effective refractive index	1.4
n_w	refractive index of water	1.33
max_{numb}	maximum number of particles	5500

*Here the input values used in the supporting video are shown

4. The origin of the two circles in the FTEMs

The purpose of the following subsection is to present a simple explanation for the origin of the circles and for the shifts of the circles presented in the manuscript. To reach that goal the mathematical description is simplified in various ways. First of all, masking of the electrical fields inside the particles is neglected. Secondly, for A_{pw} a real number is assumed so the term $\mathrm{Re}(A_{pw}e^{-j(k_x x)})$ can be written as $A_{pw}\cos(-k_x x)$. An analogous assumption is made for the spherical wave. The normalization of $E_{total,z}(x,y)$ is also neglected.

As described above the overall scattered intensity is:

$$I(x,y) \propto \left|E_{pw,z}(x) - E_{total,z}(x,y)\right|^2$$
$$= E_{pw,z}(x)^2 - 2E_{pw,z}(x)E_{total,z}(x,y) + E_{total,z}(x,y)^2.$$

The centre term $-2E_{pw,z}(x)E_{total}(x,y)$ is responsible for the shift of the circle in the Fourier transformed (FT) morphology, because the FT of the product of the two functions is the convolution of the FTs of the functions:

$$E_{pw,z}(x)E_{total,z}(x,y) \circ\!\!-\!\!\bullet\ F\{E_{pw,z}(x)\} * F\{E_{total,z}(x,y)\}$$

The field $E_{total,z}(x,y) = \sum_{i=1}^{N} E_{sw,z,i}(x - x_i, y - y_i)$ is a superposition of many spherical waves originating at different x_i, y_i. For the FT of $E_{total,z}(x,y)$, however, the shifting law[6]

$$f(x - x_0) \circ\!\!-\!\!\bullet\ F\{f(x)\} \cdot e^{-j2\pi x_0 x^{-1}}$$

can be used:

$$F\left\{\sum_{i=1}^{N} E_{sw,z,i}(x - x_i, y - y_i)\right\} = F\{E_{sw,z}(x,y)\} \cdot \sum_{i=1}^{N} e^{-j2\pi\left(x_i x^{-1} + y_i y^{-1}\right)}$$

so its FT can be understood as a product. The first term corresponds to the FT of a single spherical wave originating from $x = 0, y = 0$. The second term contains the information of the AgNP positions. Its complete mathematical discussion is beyond our scope. However, for a large number of statistically assembled particles, this factor would converge to zero. With the described alignment it will maximize the presented phenomenon.

Defining: $FA(x^{-1}, y^{-1}) = \sum_{i=1}^{N} e^{-j2\pi\left(x_i x^{-1} + y_i y^{-1}\right)}$

it can be written:

$$-2E_{pw,z}(x)E_{total,z}(x,y)) \circ\!\!-\!\!\bullet -2\, FA(x^{-1},y^{-1})(F\{E_{pw,z}(x)\} * F\{E_{sw,z}(x,y)\})$$

That means that the FT of the scattered intensity contains a convolution of the functions: $F\{E_{pw,z}(x)\}$ and $F\{E_{sw,z}(x,y)\}$.

Under the assumption described above, the first function is [6]:

$$F\{E_{pw,z}(x)\} = A_{pw}\frac{\delta\big((x^{-1}-\lambda_x^{-1})\big)+\delta\big((x^{-1}+\lambda_x^{-1})\big)}{2}$$

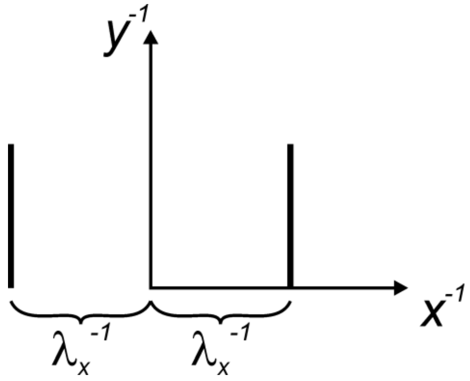

Figure S5 The Fourier Transformed plane wave. $F\{E_{pw,z}(x)\}$ consists of two Dirac pulses at $x^{-1} = -\lambda_x^{-1}$ and $x^{-1} = +\lambda_x^{-1}$.

The spherical wave $E_{sw,z}(x,y)$ propagates not only in x, but also in any direction (polar coordinate r). Moreover, the wavelength is different as it propagates in water.

Substituting $x \to r, \lambda_x^{-1} \to \lambda_w^{-1}$ the FT of $E_{sw,z}(x,y)$ results in a circle as shown in Figure S6.

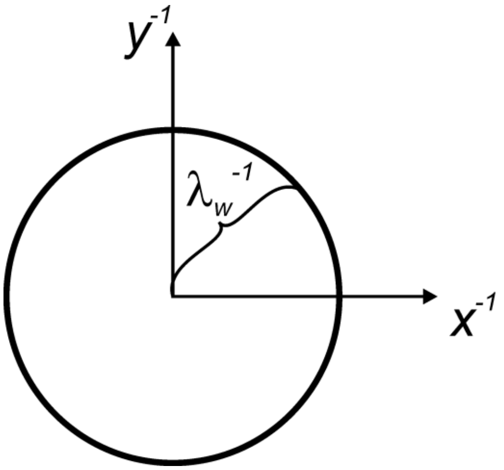

Figure S6 The Fourier Transformed spherical wave. $F\{E_{sw,z}(x,y)\}$ is represented by a circle with the centre at $x^{-1} = y^{-1} = 0$ and a radius λ_w^{-1}.

The convolution of that circle with the two Dirac pulses of $F\{E_{pw,z}(x)\}$ means a shift by $\pm\lambda_x^{-1}$.

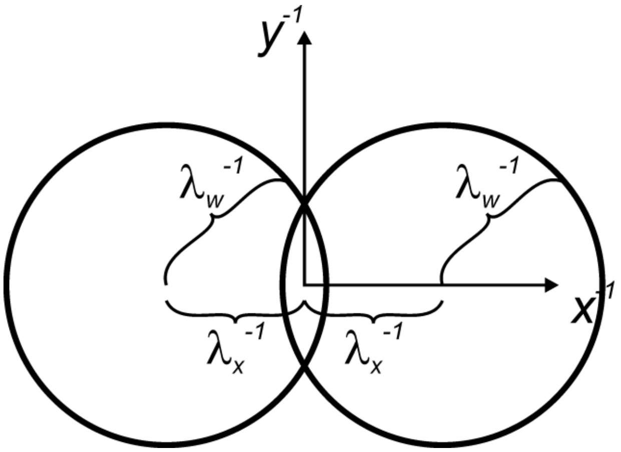

Figure S7 The Fourier Transformed product $\boldsymbol{F\{E_{pw,z}(x)\} * F\{E_{sw,z}(x,y)\}}$. The circle of $F\{E_{sw,z}(x,y)\}$ (see S6) is shifted by $\pm\lambda_x^{-1}$.

5. The supplementary videos and Python source code

The supplementary videos visualize the algorithm that describes the AgNP assembly. Figure S8 shows selected frames of supplementary video 1. With increasing number of particles (right), the visibility of the two circles in the simulated FTEM (left) also increases. Supplementary video 2 shows the corresponding electrical field. Supplementary video 3 visualizes how particles are placed by the algorithm.

The Python source code of the algorithm is included at the end of this document.

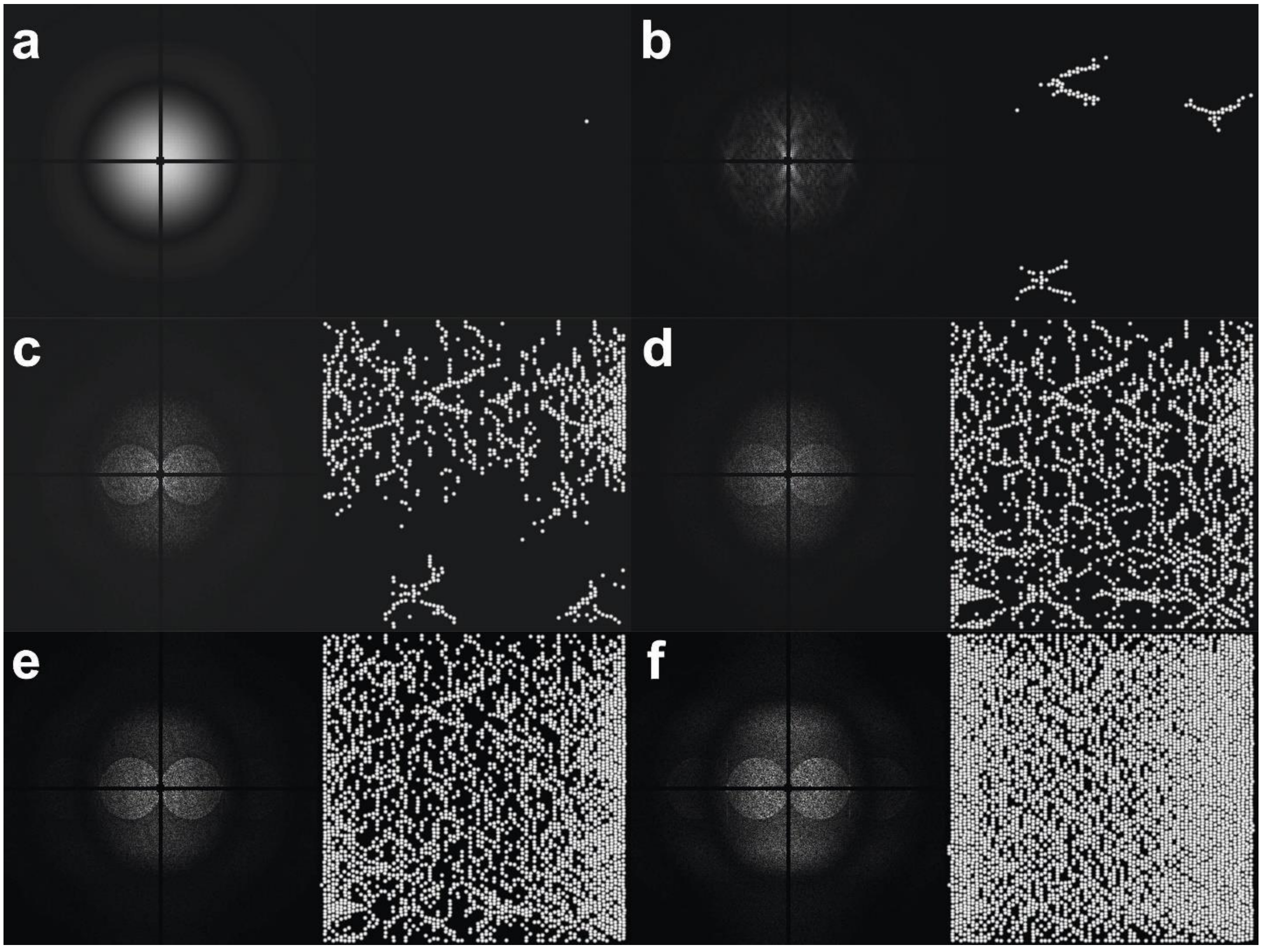

Figure S8 Selected frames of the supplementary video 1. The simulated electrom micrographs (right) and the corresponding FTs (left) are shown for **a,** 1 particle, **b,** 100 particles, **c,** 1000 particles, **d,** 2000 particles, **e,** 3000 particles and **f,** 5000 particles.

1 Saito, Y., Wang, J. J., Smith, D. A. & Batchelder, D. N. A simple chemical method for the preparation of silver surfaces for efficient SERS. *Langmuir* **18**, 2959-2961, doi:10.1021/la011554y (2002).

2 Polywka, A., Jakob, T., Stegers, L., Riedl, T. & Görrn, P. Facile Preparation of High-Performance Elastically Stretchable Interconnects. *Adv Mater* **27**, 3755-3759, doi:10.1002/adma.201501461 (2015).

3 Giannini, V. *et al.* Controlling Light Localization and Light-Matter Interactions with Nanoplasmonics. *Small* **6**, 2498-2507, doi:10.1002/smll.201001044 (2010).

4 Kelly, K. L., Coronado, E., Zhao, L. L. & Schatz, G. C. The optical properties of metal nanoparticles: The influence of size, shape, and dielectric environment. *J Phys Chem B* **107**, 668-677, doi:10.1021/jp026731y (2003).

5 Amendola, V., Bakr, O. M. & Stellacci, F. A Study of the Surface Plasmon Resonance of Silver Nanoparticles by the Discrete Dipole Approximation Method: Effect of Shape, Size, Structure, and Assembly. *Plasmonics* **5**, 85-97, doi:10.1007/s11468-009-9120-4 (2010).

6 Lücke, H. D. *Signalübertragung*. (Springer-Verlag, 1998).

Python Source Code

The following code is an extension of the algorithm explained above allowing slight particle overlap in order to reach a larger numbers of particles. Here, in the small allowed overlap region of two particles (b_i, b_j) the higher brightness is chosen instead of the sum, enabling more natural overlapping.

```
# -*- coding: utf-8 -*-
"""
Before starting, you have to create a folder 'images' in your working directory.
You need following packages to run this code:
numpy,matplotlib,scipy,skimage

The script was written with python 2.7

"""
import gc
import numpy as np
import scipy.ndimage
import matplotlib.pyplot as plt
from numpy import pi
import os
from skimage.draw import circle_perimeter_aa
gc.enable()

areal=12000
dim=int(2400)
```

```
def main():                    ############################              main
##########################
    r_particle=110.
    r_particle_px=np.around(r_particle/(areal/dim))
    w=0.016
    i_min=0.80
    A_pw=1.0
    A_sw=1.0
    lambda0=405.
    angle=70.
    angle_rad=np.deg2rad(angle)
    n_h2o=1.33
    n_eff=1.4
    err=0.
    wl_x=(((lambda0/n_eff)/np.sin(angle_rad)/(areal/dim)))
    folder=os.getcwd()+'\\images\\'
    max_numb=6000
    number_of_particles=0
    delta_x=0
    delta_y=0
    E_total=np.zeros((dim,dim),dtype=complex)
    img_dipol=np.zeros((dim,dim),dtype=complex)
    particlefield=np.zeros((dim,dim),dtype=np.float32)
    particlefield_mask=np.zeros_like(particlefield,dtype=bool)
    x_index=np.arange(-dim/2-1,dim/2-1)
    y_index=np.arange(-dim/2-1,dim/2-1)
    for x in range(0,len(x_index)):
        for y in range(0,len(y_index)):
            img_dipol[y,x]=x+(y)*1j


    def make_fft(particlefield_temp):
        particlefield_fft=np.array(particlefield_temp,copy=True)
```

```
        E_total_fft=np.fft.fft2(np.float32(particlefield_fft))
        E_total_fft[0][0]=0
        E_total_fft=np.abs(E_total_fft)
        E_total_shift=np.fft.fftshift(E_total_fft)
        E_total_shift[dim/2-2:dim/2+3]=0
        E_total_shift[::,dim/2-2:dim/2+3]=0
        E_total_shift[dim/2-5:dim/2+5,dim/2-5:dim/2+5]=0
        E_total_shift=E_total_shift[1000:1400,1000:1400]
        E_total_shift=scipy.ndimage.interpolation.zoom(E_total_shift,6)

E_total_2in1=np.concatenate((E_total_shift/np.max(E_total_shift),particlefield_fft/np.max(partic
lefield_temp)),axis=1)
        return E_total_2in1

    def E_pw(wl_x):
        E_pw=np.ones((dim,dim),dtype=np.float)
        E_pw=(np.exp(-2j*np.pi/wl_x*np.arange(0,dim)))
        return E_pw.real

    def find_place(particlefield,particlefield_mask,i_min):
        E_total[np.nonzero(particlefield_mask)]=0
        E_total_spline_temp=np.array(E_total,copy=True,dtype=np.complex64)

E_total_spline_temp=((E_total_spline_temp)/np.nanmax((E_total_spline_temp.real)))+E_pw(wl
_x).real
        E_total_spline_temp[np.nonzero(particlefield_mask)]=0

temp=np.where((E_total_spline_temp.real)**2>=i_min*np.nanmax((E_total_spline_temp.real)**
2))
        temp0=np.argsort(np.array(E_total_spline_temp[temp]))
        temp0=temp0[::-1]
        if np.shape(temp)[1]==0:
```

```
            temp=np.nanargmax((E_total_spline_temp.real)**2)
            temp=np.unravel_index(temp,(dim,dim))
            temp=np.array([temp[0],temp[1]])
            return
(np.array([temp[0],temp[0]]),np.array([temp[1],temp[1]])),np.count_nonzero(particlefield)/dim**
2.
        belegung=np.count_nonzero(particlefield_mask)/dim**2.
        return (np.array(temp[0][temp0]),np.array(temp[1][temp0])), belegung

    def E_sw(x_i,y_i,r_particle,n,angle,img_sw,phi):
        angle=np.deg2rad(angle)
        k_vac=2*np.pi/(405./(areal/dim))
        k_matter=k_vac*n
        r_particle=np.around(r_particle/(areal/dim))
        img_sw0=img_sw-x_i-y_i*1j
        img_sw0=np.where((np.abs(img_sw0)>r_particle),np.abs(img_sw0),r_particle+1)
        rho=(np.abs(img_sw0))*(k_matter)
        E_field=(np.exp(1j*(rho+phi))*r_particle/(np.abs(img_sw0)))
        return E_field.real

    def b_i(x_i,y_i,r_particle,particlefield_mask,particlefield):
        r_particle=np.around(r_particle/(areal/dim))
        particlefield_newp=np.zeros_like(particlefield)
        k_part=1./(4.*r_particle)
        AA_elli=np.linspace(0.,r_particle,(r_particle+1))
        w0=w*(areal/dim)
        for i in AA_elli:
            color0=(1.-np.sin(2.*pi*k_part*i))*np.exp(w0*i)
            pts=circle_perimeter_aa(x_i,y_i,(int(i)))
            particlefield_newp[pts[1],pts[0]]=color0
            particlefield_mask[pts[1],pts[0]]=True
        pos_newp=np.nonzero(particlefield_newp)
```

```
        if np.any(particlefield[pos_newp])==1:
            posres=np.nonzero(particlefield[pos_newp])

gap=np.where(particlefield[pos_newp[0][posres],pos_newp[1][posres]]>=particlefield_newp[pos
_newp[0][posres],pos_newp[1][posres]],particlefield[pos_newp[0][posres],pos_newp[1][posres]]
,particlefield_newp[pos_newp[0][posres],pos_newp[1][posres]])
            particlefield[pos_newp]=(particlefield[pos_newp]+particlefield_newp[pos_newp])
            particlefield[pos_newp[0][posres],pos_newp[1][posres]]=gap
        else:
            particlefield[pos_newp]=particlefield_newp[pos_newp]
        return particlefield

    particlefield_mask[0:dim,0:int(1.5*r_particle_px)]=True
    particlefield_mask[0:dim,dim-int(r_particle_px*1.5)+1:dim]=True
    particlefield_mask[0:int(r_particle_px*1.5),0:dim]=True
    particlefield_mask[dim-int(r_particle_px*1.5)+1:dim,0:dim]=True
    for i in range(0,3):
        x_start=np.random.randint(int(r_particle_px*1.5),dim-int(r_particle_px*1.5))
        y_start=np.random.randint(int(r_particle_px*1.5),dim-int(r_particle_px*1.5))
        phi=((2.*np.pi/wl_x*(x_start)))
        E_total=E_total-E_sw(x_start,y_start,r_particle,n_h2o,90,img_dipol,phi)
        particlefield=b_i(x_start,y_start,r_particle,particlefield_mask,particlefield)
        number_of_particles=number_of_particles+1

    while number_of_particles<max_numb+1:
        next_particle,belegung=find_place(particlefield,particlefield_mask,i_min)
        for next_p in np.nditer(next_particle):
            if particlefield_mask[next_p[0],next_p[1]]==False:
                if err!=0:
                    delta_x=int(np.random.normal(0,err))
                    delta_y=int(np.random.normal(0,err))
                phi=(2.*np.pi/wl_x*(next_p[1]))#
```

```
E_h2o=A_sw*E_sw(next_p[1]+delta_x,next_p[0]+delta_y,r_particle,n_h2o,90,img_dipol,phi)
        E_total=A_pw*E_total-E_h2o

particlefield=b_i(next_p[1]+delta_x,next_p[0]+delta_y,r_particle,particlefield_mask,particlefield
)
        number_of_particles=number_of_particles+1
        if number_of_particles%10==False:
          E_total[np.nonzero(particlefield_mask)]=0
          E_total_2in1=make_fft(particlefield)

(plt.imsave(folder+'_areal'+str(areal/1000.)+'um_'+str(r_particle)+str(angle)+'grad_i_min'+str(i_
min)+'_A_pw'+str(A_pw)+'_'+str(number_of_particles)+'.jpg',E_total_2in1,cmap='gray'))
        gc.collect()


main()
```
